# Investigating the Local Bonding Structure of Amorphous Zinc Tin Oxide to Elucidate the Effect of Altering the Intercation Ratio

**DOI:** 10.1021/acs.jpcc.4c04225

**Published:** 2024-09-20

**Authors:** Peter J. Callaghan, Karsten Fleischer, David Caffrey, Kuanysh Zhussupbekov, Stuart Ansell, Yurii K. Gun’ko, Igor V. Shvets, Ainur Zhussupbekova

**Affiliations:** † School of Physics and Centre for Research on Adaptive Nanostructures and Nanodevices (CRANN), Trinity College Dublin, Dublin 2, Ireland; ‡ School of Physical Sciences, Dublin City University, Dublin 9, Ireland; § School of Chemistry, Trinity College Dublin, Dublin 2, Ireland; ∥ MAX IV Laboratory, Lund 22100, Sweden

## Abstract

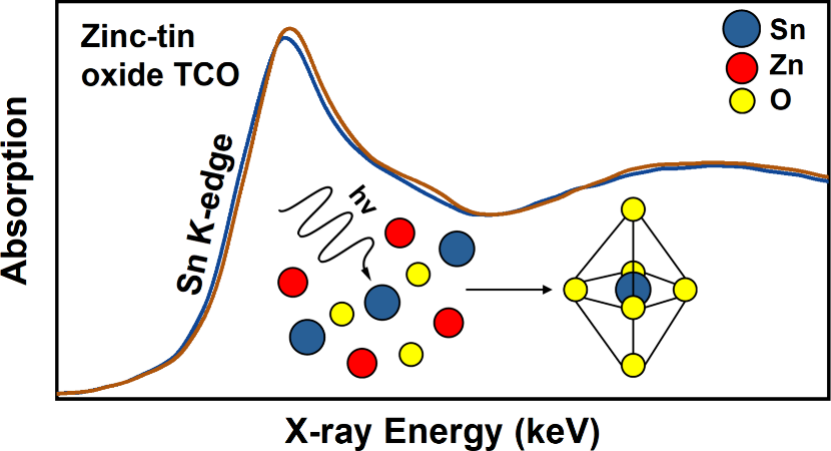

In this paper, the local bonding structure in amorphous zinc tin oxide (a-ZTO) is probed using a combination of XANES and EXAFS techniques at the Zn and Sn K-edges to gain insight into charge carrier generation in the material. a-ZTO is prepared using two growth methods; spray pyrolysis and magnetron sputtering. It is seen that a-ZTO grown by magnetron sputtering shows no changes in the chemical environment as the cation ratio is varied; meanwhile, XANES analysis of spray pyrolysis grown samples shows alterations to spectra likely due to the effects caused by different precursors. Although a slight shift in Sn–O bond length is visible between magnetron sputtered and spray grown samples, no correlation could be discerned between bond length and variation in cation ratio. It is concluded that a-ZTO, while amorphous over longer ranges, is locally composed of ZnO and SnO_2_ “building blocks”. An alteration in the cation ratio changes the hybridization at the conduction band minimum, resulting in the observed variation in the mobility, charge carrier concentration, and bandgap.

## 1 Introduction

Zinc tin oxide (ZTO) is an exciting alternative to Sn-doped indium oxide (ITO) and indium gallium zinc oxide (IGZO) that have dominated the transparent conducting oxide (TCO) market for thin film transistors in recent years.^1−6^ While materials such as indium zinc oxide (IZO) have shown mobilities and charge carrier concentrations as high as μ = 54 cm^2^/V·s and *n* = 1.3 × 10^20^ cm^−3^, respectively,^7^ they show a reliance on the presence of In to achieve these high conductivities. The issues with the use of In are numerous, but chief among them are concerns surrounding cost and sustainability,^8^ low flexibility,^5^ and health concerns.^9−11^ ZTO is entirely composed of low-cost and sustainable elements^12^ and shows excellent properties for a TCO, namely high conductivity and low absorption in the visible region.^13,14^ Further, ZTO can be deposited in an amorphous form while retaining impressive conductivity and transparency values without the need for postgrowth treatment.^15,16^ This high performance in the amorphous form means ZTO can be deposited at lower temperatures, making it compatible with flexible plastic substrates such as polyethylene terephthalate (PET).^5,17^ As such, ZTO has already seen varied use in applications such as organic LEDs,^18^ gas sensors,^19,20^ thin film transistors,^21,22^ and photovoltaics.^23−25^

As an amorphous ternary oxide, the behavior of amorphous zinc tin oxide (a-ZTO) can be complex and difficult to understand. Previous studies by Zhussupbekova et al. have shown that when one varies the cation/cation ratio in a-ZTO thin films, this can have a corresponding effect on the charge carrier concentration, electron mobility, and absorption coefficient—all important parameters for a TCO.^26,27^ It is hypothesized that as one varies the cation ratio in a-ZTO, this may result in changing the polymorph of a-ZTO present in the material, namely the predicted ZnSn_2_O_5_ and ZnSn_3_O_7_.^26,28,29^ As these polymorphs may possess alterations to the local chemical environment or bonding structure, this may affect the formation energy of defects leading to an observed difference in charge carrier concentration and overall material conductivity. Clarity around the local bonding structure as the zinc/tin ratio is varied would open opportunities for computational modeling of the material, which in turn could elucidate the doping mechanisms of the ternary oxide material as well as help identify the optimal growth conditions for maximizing mobility and charge carrier concentrations for device applications.

In this paper, we employ a combination of X-ray absorption near edge structure (XANES) and extended X-ray absorption fine structure (EXAFS) to study the effect of cation ratio change on local bond structure and physical properties of films grown by magnetron sputtering and spray pyrolysis. Both techniques examine the modulation of the X-ray absorption coefficient (μ) with incident X-ray photon energy. In XANES this is typically used to give information on the chemical environment and oxidation state of the absorbing atom, while in EXAFS, in collaboration with FEFF9 ab initio fitting software, it can be correlated to the local bonding environment of the absorbing atom.

## 2 Method

### 2.1 Sample Growth

Amorphous ZTO thin films were prepared by nonreactive RF magnetron sputtering and spray pyrolysis. The sputtered films were grown under an Ar atmosphere on quartz glass substrates at a pressure of 1 × 10^–3^ mbar. Depositions were performed using custom single ZTO targets prepared by Kurt J. Lesker with preset Zn/Sn ratios of 30/70 (MAG1) and 50/50 (MAG3). A power density of 15 W/in. was applied to the MAG1 target, while a power density of 21.5 W/in. was applied to the MAG3 target, in both cases achieving a deposition rate of 2 nm/min. A combination of both targets was used to achieve a 40/60 ratio (MAG2), with a deposition rate of 4 nm/min. Prior to deposition, the substrate was heated to 300 °C, and a target presputter was performed to clean the surface of the sputtering target. A reference sample of SnO_2_ was also prepared via magnetron sputtering using a SnO_2_ target. Spray pyrolysis synthesis was performed using the system and deposition conditions described in the previous work.^27^ Four different combinations of precursors with Zn/Sn solution ratios of 50/50 were studied: zinc acetate dehydrate + tin(II) 2-ethylehexanoate (SP1), zinc acetylacetonate hydrate + tin(II) 2-ethylehexanoate (SP2), zinc acetylacetonate hydrate + tin chloride (SP3), and zinc chloride + tin(II) 2-ethylehexanoate (SP4).

### 2.2 Thin Film Characterization

The Zn/Sn ratio in the thin films was confirmed via X-ray photoelectron spectroscopy (XPS). Magnetron samples were transferred *in vacuo* using a vacuum suitcase,^30^ and spray samples were transferred in air and subsequently sputtered clean.^27^ Core level scans were performed by using an Omicron MultiProbe XPS system equipped with a monochromatic Al Kα source (XM 1000, 1486.7 eV). The instrument has a base pressure of 5 × 10^–11^ mbar and an instrumental resolution of 0.6 eV. The conductivity, Hall mobility, and charge carrier concentrations of the samples were measured using a four-point probe arrangement and an 800 mT electromagnet. Electrical connections in the van der Pauw configuration were made using silver wires that were adhered to each corner of the sample with silver paint. Grazing incidence X-ray diffraction (GIXRD) measurements were performed with a Bruker D8 Advance X-ray diffractometer using an unmonochromated Cu Kα source (1.54 Å) at 40 kV and 40 mA through a 0.1 mm slit at an angle of 15°–70°.

### 2.3 X-ray Absorption Spectroscopy

X-ray absorption spectroscopy (XAS) was performed at the BALDER beamline at the MAX IV synchrotron facility in Lund, Sweden. In this study, we perform XAS at room temperature at the Zn and Sn K-edges, which occur at 9.66 and 29.2 keV, respectively. Crucially both XANES and EXAFS are independent of the crystalline nature of the material, making them well-suited to examine the bonding structure of a-ZTO thin films. The Zn K-edge at 9.66 keV and the Sn K-edge at 29.2 keV were chosen for this study, as the K-edge transitions are more sensitive to changes in coordination and local geometry required for local bond arrangement studies. The necessary wavelength was selected by using a Si{111} double crystal monochromator. The XANES and EXAFS spectra measured at the Sn edge were collected in a transmission configuration, while XANES data at the Zn edge were collected using a PIPS detector in fluorescence detection mode. Multiple scans were recorded for a given spectra and subsequently merged in order to improve the signal-to-noise ratio. XANES data were treated with a FFT smoothing filter with a box size of 5. EXAFS data were treated with a second-order Savitzky–Golay smoothing function with a box size of 11.

Data were analyzed using the Larix software created by Matthew Newville.^31^ In order to normalize the X-ray absorbance factor (μ(*E*)), XAS spectra were fitted with a first-order Victoreen pre-edge function and a polynomial post-edge function. In addition, a smooth atomic signal (*R*_bkg_) was subtracted from EXAFS data in order to subtract the contribution of the absorbing atom. After data normalization EXAFS data were converted from energy space to *k*-space using the relationship , where *k* is the electron wavenumber, *m* is the mass of an electron, *E*_0_ is the K-edge absorption edge for the spectra, and *ℏ* is the reduced Planck constant. EXAFS spectra were multiplied by *k*^2^ to compensate for the amplitude decay at higher *k* values. To achieve information on the shells surrounding the absorbing Sn atom, EXAFS spectra were multiplied by a Kaiser–Bessel window function and then Fourier transformed into *R*-space in the range 1–8 Å. FEFF9 ab initio calculations were used to simulate the recorded data and extract bond length and Debye–Waller factors.^32^

## 3 Results

### 3.1 Physical Characterization

High quality a-ZTO thin films with varying Zn/Sn ratios were produced by nonreactive RF magnetron sputtering; the properties of the thin films are summarized in Table 1. One should be aware that the target or precursor ratio does not always directly transfer to the deposited thin films; thus, in order to verify the cation composition of the thin films, the samples were transferred to an Omicron Multiprobe XPS system. Further details of the procedure are available in previously published work.^27,30^ The films showed excellent conductivity values and low absorption in the visible range, as seen in Figure S1 of the Supporting Information. Notably it is seen that by varying the cation ratio, properties such as charge carrier concentration (*n*) and Hall mobility (μ_Hall_) can be effectively controlled. This behavior is in agreement with previous studies on a-ZTO, which in addition show that the bandgap of the material can also be tuned via the intercation ratio.^26^

**Table 1:** a-ZTO Thin Films Were Prepared at Various Zn:Sn Ratios by Magnetron Sputtering and via Spray Pyrolysis Using a Range of Precursors (Cation Compositions Were Confirmed by XPS Analysis)

sample name	film Zn/Sn ratio (%)	σ (S/cm)	μ_Hall_ (cm^2^/V·s)	*n* (cm^–3^)
MAG1	20:80	228	17.4 ± 1.7	(8.2 ± 0.8) × 10^19^
MAG2	27:73	131	16.0 ± 1.6	(5.1 ± 0.5) × 10^19^
MAG3	41:59	76	19.5 ± 2.0	(2.4 ± 0.2) × 10^19^
SP2	43:57	180	8.2 ± 0.9	(1.4 ± 0.1) × 10^20^
SP3	31:69	24	3.2 ± 0.4	(4.7 ± 0.5) × 10^19^
SP4	23:77	304	10.2 ± 1.1	(1.9 ± 0.2) × 10^20^

### 3.2 XANES of the Zn K-Edge

**Figure 1:**
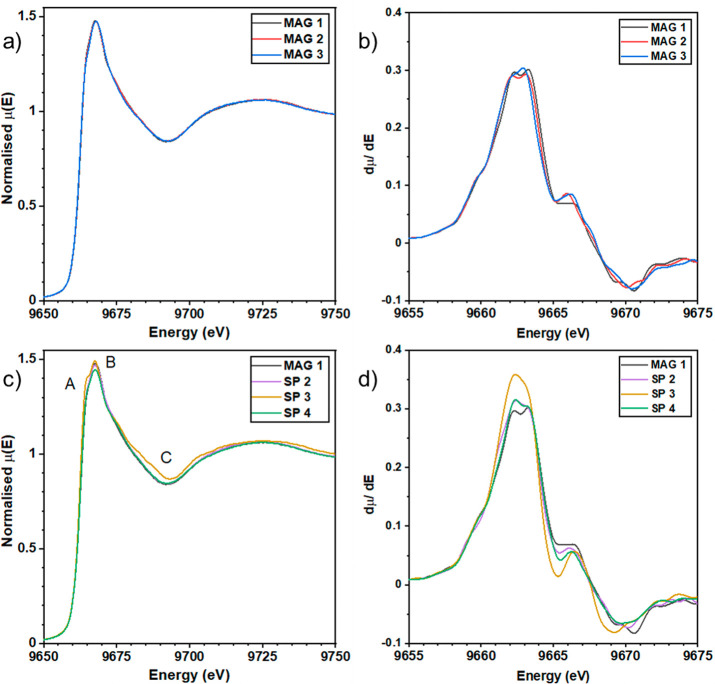
Normalized XANES spectra at the Zn K-edge of (a) magnetron grown a-ZTO and (b) dμ/d*E* of magnetron grown a-ZTO. Normalized XANES spectra of (c) spray pyrolysis grown a-ZTO and (d) dμ/d*E* of spray pyrolysis grown a-ZTO. Small variations are noticeable in the line shape of spray pyrolysis grown samples, suggesting slight differences in the chemical environment.

To probe changes in the local chemical and bonding environment as the cation ratio was varied, XAS was measured and normalized at the Zn K-edge and Sn K-edge, respectively. XANES relies on there being a finite number of chemical environments in which an atom may exist, and the XANES spectra of these different chemical environments are consistent within a functional group and easily distinguishable from one another. The Zn K-edge captures transitions from the Zn 1s orbital to empty Zn 4p orbitals in the conduction band. The XANES spectra of magnetron grown samples are depicted in Figure 1a. The spectra are characterized by a relatively broad main peak at 9668 eV; this peak broadness is due to the amorphous structure of the thin films and is characteristic of a-ZTO.^33,34^ It is interesting to note that the peak shape remains consistent across all magnetron grown samples with no shift in the main absorption edge. As differences in μ(*E*) can often be subtle and difficult to discern, the first derivative dμ/d*E*, shown in Figure 1b, was also examined to more closely evaluate any possible changes in line shape. A doublet peak with a subtle shift toward higher binding energy as the Sn cation concentration is increased is observed.

For comparison to the magnetron grown a-ZTO, samples of a-ZTO prepared via spray pyrolysis were also examined and are shown in Figure 1c. Spray pyrolysis being a chemical vapor deposition method displays different growth mechanisms compared to magnetron sputtering; thus, a-ZTO prepared using this technique may serve as a suitable comparison to any changes observed in magnetron grown a-ZTO. There are clear differences between the spectra of spray grown a-ZTO, most visible at points A and C of SP3, with the shoulder seen at 9665 eV typical of crystalline ZnO.^33^ The dμ/d*E* of spray grown samples is shown in Figure 1d with a magnetron grown sample included for visual reference. The most noticeable changes are seen in SP3, where the even doublet present in magnetron grown a-ZTO is lost and replaced with a dominant peak at 9662 eV. One notes a similar shift in maximum peak intensity (toward 9662 eV) in SP2 and SP4 suggesting a slight shift to a more crystalline ZnO environment.

### 3.3 XANES at the Sn K-Edge

**Figure 2:**
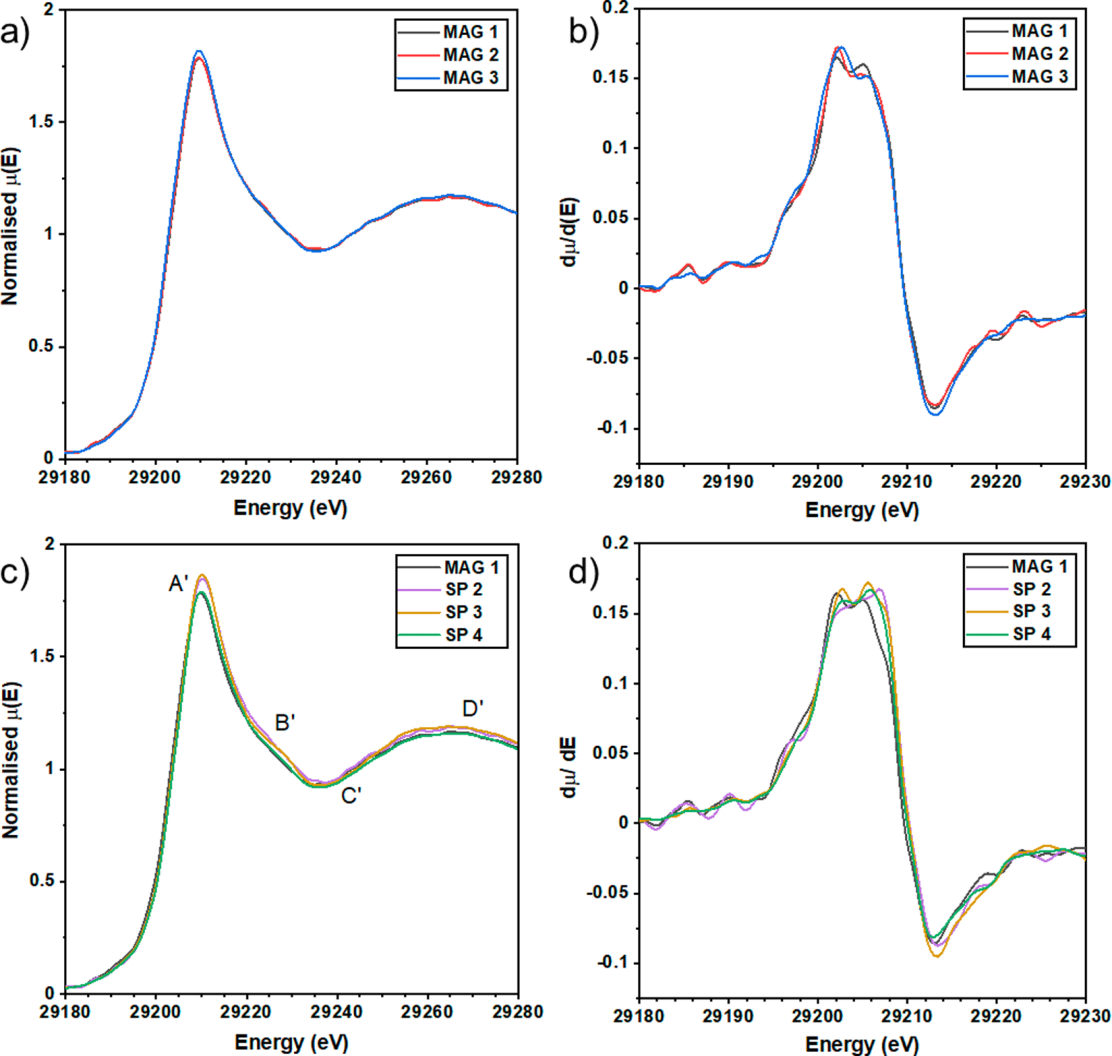
Normalized XANES spectra at the Sn K-edge for (a) magnetron grown a-ZTO and (b) dμ/d*E* of magnetron grown a-ZTO. Also, normalized XANES spectra of (c) spray pyrolysis grown a-ZTO and (d) dμ/d*E* of spray pyrolysis grown a-ZTO. A blue-shift in peak maxima is observed in samples SP2, SP3, and SP4.

XANES spectra from the Sn K-edge for magnetron grown a-ZTO are shown in Figure 2a. This is dominated by transition from the Sn 1s orbital to the empty Sn 5p orbital in the conduction band. Similar to the Zn XANES spectra, the single broad peak at 29210 eV is typical of a-ZTO.^33^ Within the magnetron grown samples, one notices the small increase in the white line intensity of MAG3; however, the peak shape and absorption edge position remain unchanged. Examining the dμ/d*E* for magnetron grown a-ZTO in Figure 2b, there are no discernible differences between the spectra of magnetron grown a-ZTO within the noise limit. All samples exhibit a relatively broad peak with a sharp maximum at 29202 eV.

There are several points of note when comparing the spray pyrolysis grown a-ZTO shown in Figure 2c. First, SP4 is very alike the magnetron grown a-ZTO XANES spectra, suggesting extremely similar chemical environments. Second, samples SP2 and SP3 show a broader absorption peak and differences in the post-edge region most noticeable at the crossover points of points B′, C′, and D′. Finally, there is a slight blue-shift in the absorption edge of spray grown samples; this is shown more clearly in Figure 2d. Examining the dμ/d*E* of spray grown ZTO in Figure 2d, a feature at 29202 eV is still observed in all spectra; however, there is also a noticeable increase in intensity of the feature at 29206 eV in SP3 and SP4. This feature is continued to be blue-shifted in SP2, with the maximum absorption seen at 29207 eV.

From XANES spectroscopy at the respective Zn and Sn K-edges it is evident that there is no change in the chemical environment or oxidation state in magnetron grown a-ZTO. This is shown in both μ(*E*) and dμ/d*E* of the Zn and Sn edge, where there is no discernible difference between spectra within the noise limit. In the case of spray grown a-ZTO, different growth precursors result in clear differences in the μ(*E*) and dμ/d*E*, particularly in SP3 at the Zn edge. This is likely due to small amounts of unreacted precursor being present in the sample, which affect the local chemical environment of the cations.

### 3.4 EXAFS at the Sn K-Edge

In addition to the analysis of the near-edge regime shown above, EXAFS measurements of the extended post-edge region at the Sn K-edge were also recorded to probe the local bonding environment in a-ZTO. These spectra were normalized, and a μ_0_ background function was subtracted (*R*_bkg_ = 0.8) from all spectra. Subsequently, the *k*^2^χ(*k*) was calculated for both magnetron grown and spray pyrolysis grown a-ZTO. To extract quantitative information about the bond type, bond distance and structural disorder in a-ZTO the χ(*R*) spectra were fitted using the EXAFS equation (eq 1) and FEFF9 ab initio calculations in the Larix software.
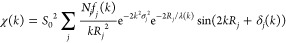
1where *j* indicates shells of like atoms, *S*_0_^2^ is the passive electron reduction factor, *N*_*j*_ is the coordination number of an atom in the *j*th shell, *k* is the photoelectron wavenumber, *R*_*j*_ is the distance to the neighboring atom, σ_*j*_^2^ is the Debye–Waller factor, and λ(*k*) is the electron mean free path. There is also an induced scattering amplitude *f*_*j*_(*k*) and phase shift δ_*j*_(*k*) that are dependent on the atomic number of the scattering atom.

**Figure 3:**
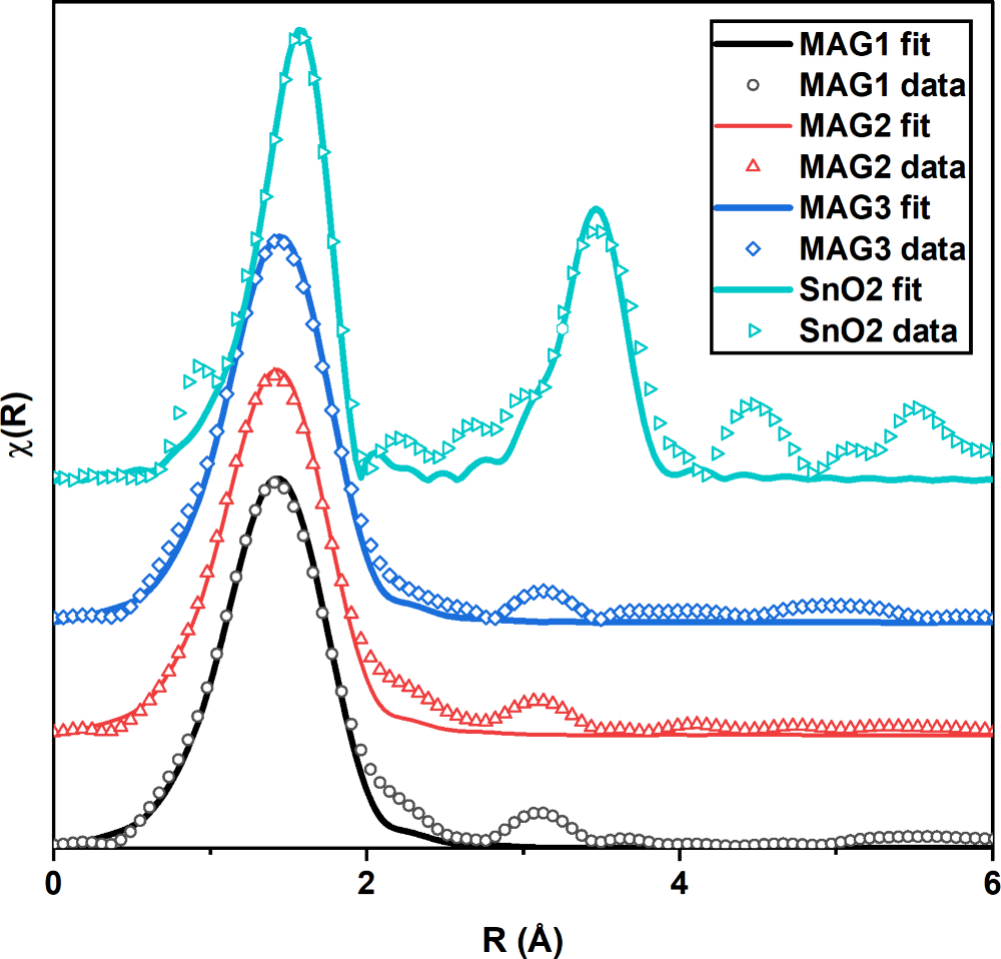
χ(*R*) spectra calculated from EXAFS measurements at the Sn K-edge and corresponding FEFF9 fits for magnetron grown a-ZTO and reference SnO_2_. Because of the amorphous nature of the a-ZTO, a higher order Sn–Sn bond is only observed for SnO_2_.

The first peak in χ(*R*) spectra shown in Figure 3 comes from the first nearest-neighbor (1NN) shell of atoms to the absorbing atom. It is possible for higher order shells to appear in χ(*R*), but due to the amorphous nature of the a-ZTO thin films, the higher order signal is quickly attenuated in all samples due to the lack of long-range order. This agrees with XRD characterization shown in Figure S2 of the Supporting Information. Because of this strong attenuation in the χ(*R*) signal, only the first shell of a-ZTO was fitted to in FEFF9 calculations, which in this case relates to Sn–O bonds. In addition, as we consider only 1NN atoms, only single scattering paths for the photoelectron are included in the fitting. A small peak feature is visible at 3.2 Å; however, it is too small to be able get a meaningful fit due to the presence of multiple photoelectron scattering paths in this region.

The starting inputs for the Sn–O path are based on rutile SnO_2_, space group (*P*42/*mnm*).^35^ The χ(*R*) spectra and fits are shown in Figure 3, and fit parameters are shown in Table 2. A small shift of 0.02 Å is seen in the Sn–O bond length between sputtered a-ZTO samples and the SnO_2_ reference sample. This SnO_2_ reference thin film, also prepared via magnetron sputtering, shows good agreement with the Sn–O bond distance recorded in the literature.^35−37^

**Table 2:** FEFF9 Fit Parameters after Fitting to the First Shell, Where N Is the Sn Coordination Number, *R* Is the Sn–O Bond Distance, and σ^2^ Is the Debye–Waller Factor

sample name	*NS*_0_^2^	*R* (Å)	σ^2^
MAG1	6.1 ± 0.5	2.031 ± 0.009	0.008 ± 0.002
MAG2	6.1 ± 0.6	2.031 ± 0.009	0.008 ± 0.002
MAG3	6.0 ± 0.5	2.035 ± 0.009	0.007 ± 0.002
SP1	5.8 ± 0.6	2.042 ± 0.010	0.007 ± 0.002
SP2	6.1 ± 0.6	2.029 ± 0.010	0.008 ± 0.002
SP3	6.0 ± 0.7	2.041 ± 0.011	0.005 ± 0.002
SP4	5.7 ± 0.6	2.042 ± 0.011	0.006 ± 0.001
SnO_2_	5.6 ± 0.6	2.053 ± 0.007	0.003 ± 0.001

Analysis of the χ(*R*) spectra shows no long-range order in any of the a-ZTO samples and hence discounts the formation of a-ZTO polymorphs at a local, next-nearest-neighbor scale as had previously been suggested.^26,28,29^ Trejo et al. propose that the Sn atoms in a-ZTO form distorted SnO_2_ polyhedra.^38^ This is reflected in χ(*R*) data shown in Figure 3 and Table 2 where the Sn atoms show a pronounced Sn–O shell and possess an approximately octahedral coordination. We attribute the large variation in Sn coordination number due to dangling bonds whose concentration increases as the amorphous nature of the films increases.

We propose that as there is no long-range order in the films, these distorted SnO_2_ octahedra, along with distorted ZnO tetrahedra,^34^ act as “building blocks”, which are randomly arranged throughout the entire material. As one changes the intercation ratio in the thin films, one effectively changes the ratio of building blocks within the material. The conduction band minimum (CBM) of a-ZTO is known to be a mixture of Zn 4s and Sn 5s orbitals hybridized with O 2p orbitals.^34^ Therefore, by altering the intercation ratio, one will affect the strength of hybridization at the CBM and thus affect the properties that are dependent on the electronic structure at the CBM such as electron mobility and bandgap.^26,38^

Our results confirm that the chemical bonding environment within a-ZTO does not vary greatly as the cation ratio is altered, showing no evidence for the presence of a-ZTO polymorphs. EXAFS at the Sn K-edge points toward approximately octahedral next-nearest-neighbor geometry in all measured a-ZTO samples, with no long-range order present. In addition, it is observed that varying the deposition method has very little impact on the line shape of the spectra. Furthermore, the spectra we see correlate well with previous XAS studies on ALD grown a-ZTO.^33^ The next step in this research is EXAFS at the Zn K-edge to confirm the tetrahedral bonding coordination hypothesized. Additionally, our results suggest that the mechanism responsible for the variation in conductivity values is smaller than the next-nearest-neighbor bond length that is visible from XAS, pointing toward the possible involvement of point defects. As point defects are extremely difficult to detect experimentally, computational methods may be necessary to confirm their contribution. Thus, a DFT computational study of a-ZTO to uncover the defect mechanism responsible for the variation in conductivity and confirm the optimal electrical composition of a-ZTO for electrical performance would be beneficial. In this regard, our results may prove pivotal as they elucidate the chemical and bonding environment present for Zn and Sn atoms in the material which is necessary for computational modelling.

## 4 Conclusion

Through a combination of XANES and EXAFS at the Zn and Sn K-edges, the local bonding environment in a-ZTO was examined. While varying the intercation ratio in the thin films, no changes in the chemical environment were observed for magnetron grown films while subtle differences were observed in spray pyrolysis samples, likely caused by the chemical precursors used during sample growth affecting the reaction pathways of the SnO_2_ and ZnO building blocks. EXAFS fitting at the Sn edge shows coordination numbers consistent with distorted SnO_2_ octahedra and a small decrease in Sn–O bond length for magnetron grown a-ZTO when compared to pure SnO_2_. We observe no evidence of ZnSn_2_O_5_ or ZnSn_3_O_7_ polymorphs in the form of discernible chemical environmental changes or next-nearest-neighbor scattering pathways. Rather, this result supports the theory that a-ZTO is composed of “building blocks” of ZnO and SnO_2_ polyhedra. We conclude that the observed changes in mobility and bandgap are likely derived from alterations in the level of orbital hybridization that occurs at the conduction band minimum when the Zn/Sn ratio is varied.

## Supporting Information Available

Optical measurements showing the absorption of the magnetron grown a-ZTO films in the visible light region and grazing incidence XRD confirming all samples present an amorphous signal (PDF)
