# Supplementary material for: Investigating the Local Bonding Structure of Amorphous Zinc Tin Oxide to Elucidate the Effect of Altering the Intercation Ratio
Source: J Phys Chem C Nanomater Interfaces. 2024 Sep 20;128(39):16733–9. doi: 10.1021/acs.jpcc.4c04225 (PMC11457223; doi:10.1021/acs.jpcc.4c04225)
Supplement: Supplementary file 1 — jp4c04225_si_001.pdf [file jp4c04225_si_001.pdf]

Investigating the local bonding structure of amorphous zinc tin oxide to elucidate the effect of altering the inter-cation ratio

Peter J. Callaghan,[*,†] Karsten Fleischer,[†] David Caffrey,[†] Kuanysh Zhussupbekov,[¶] Stuart Ansell,[§] Yurii K. Gun'ko,[¶] Igor V. Shvets,[†] and Ainur Zhussupbekova[*,¶]

†*School of Physics and Centre for Research on Adaptive Nanostructures and Nanodevices (CRANN), Trinity College Dublin, Dublin 2, Ireland*

‡*School of Physical Sciences, Dublin City University, Dublin 9, Ireland*

¶*School of Chemistry, Trinity College Dublin, Dublin 2, Ireland*

§*MAX IV Laboratory, Lund, 22100, Sweden*

E-mail: pcallagh@tcd.ie; zhussupa@tcd.ie

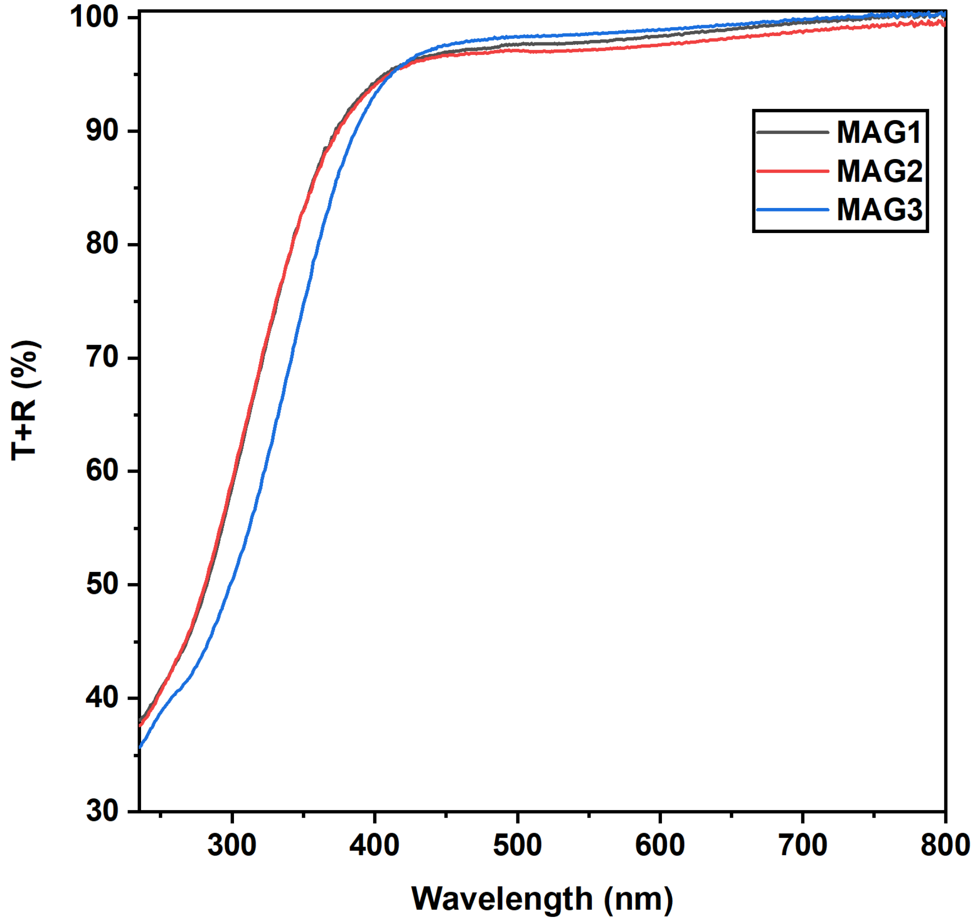

Figure S1: A combined transmission (T) and reflectance (R) spectra for the magnetron sputtered a-ZTO shows < 5% absorption until below 400 nm.

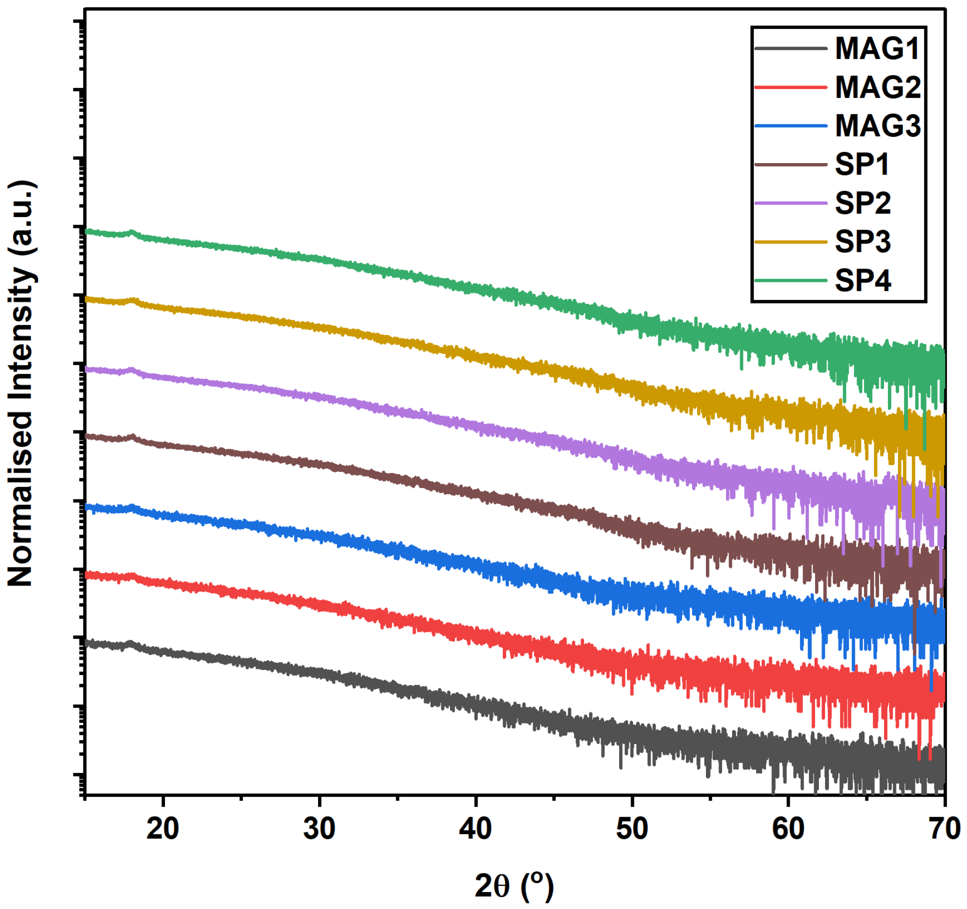

Figure S2: The amorphous nature of the thin films was confirmed via Grazing Incidence XRD (GIXRD) which show only a small peak at 17.9 ° attributed to the quartz sample holder used.
